# Comparison of two Borg exertion scales for monitoring exercise intensity in able-bodied participants, and those with paraplegia and tetraplegia

**DOI:** 10.1038/s41393-021-00642-4

**Published:** 2021-05-26

**Authors:** Michael J. Hutchinson, Ingrid Kouwijzer, Sonja de Groot, Victoria L. Goosey-Tolfrey

**Affiliations:** 1 Peter Harrison Centre for Disability Sport, School of Sport, Exercise and Health Sciences, Loughborough University, Loughborough, UK; 2 Research and Development, Heliomare Rehabilitation Center, Wijk aan Zee, The Netherlands; 3 Amsterdam Rehabilitation Research Center | Reade, Amsterdam, The Netherlands; 4 University of Groningen, University Medical Center Groningen, Center for Human Movement Sciences, Groningen, The Netherlands; 5 Department of Human Movement Sciences, Faculty of Behavioural and Movement Sciences, Vrije Universiteit Amsterdam, Amsterdam, The Netherlands

**Keywords:** Physiology, Disease prevention

## Abstract

**Study design:**

Cross-sectional cohort study.

**Objectives:**

To compare ratings of perceived exertion (RPE) on Borg’s 6–20 RPE scale and Category Ratio 10 (CR10) in able-bodied (AB) participants during upper and lower body exercise, and recreationally active participants with paraplegia (PARA) and athletes with tetraplegia (TETRA) during upper body exercise only.

**Setting:**

University and rehabilitation centre-based laboratories in UK and Netherlands.

**Methods:**

Twenty-four participants were equally split between AB, PARA, and TETRA. AB performed maximal tests using cycle (AB-CYC) and handcycle (AB-HC) ergometry. PARA and TETRA performed maximal handcycle and wheelchair propulsion tests, respectively. Oxygen uptake (V̇O_2_) and blood lactate concentration were monitored throughout. RPE was rated each stage on Borg’s RPE scale and CR10. Thresholds were identified according to log-V̇O_2_ plotted against log-blood lactate (LT_1_), and 1.5 mmol L^−1^ greater than LT_1_ (LT_2_).

**Results:**

RPE from both scales were best fit against each other using a quadratic model, with high goodness of fit between scales that was independent of exercise mode and participant group (range *R*^2^: 0.965–0.970, *P* < 0.005). Though percentage peak V̇O_2_ was significantly greater in TETRA (*P* < 0.005), there was no difference in RPE at LT_1_ or LT_2_ between groups on Borg’s RPE scale or CR10.

**Conclusion:**

Strong association between Borg’s RPE scale and CR10 suggests they can be used interchangeably. RPE at lactate thresholds were independent of mode of exercise and level of spinal cord injury. However, inter-individual variation precludes from making firm recommendations about using RPE for prescribing homogenous exercise intensity.

## Introduction

Intensity is a fundamental component of any form of exercise prescription. For athletes, this could be to maximise specific adaptations to training, leading to increased performance. For the wider population, the goal may be to improve a myriad of physical and mental health conditions [1, 2]. To account for inter-individual variance in physical function, exercise intensity is often expressed in relative terms with the aim of producing homogenous stimuli between people [3]. There remains, however, debate as to the method for how to prescribe the relative intensity [3].

One method, seemingly favoured by exercise guidelines for able-bodied (AB) [4] and adults with spinal cord injury (SCI) [5], is to use a percentage of peak oxygen uptake (%V̇O_2peak_) and heart rate (%HR_peak_) with boundaries defining the “moderate” or “vigorous” intensity that such guidelines recommend. An alternative is to use metabolic thresholds, such as the lactate threshold (LT_1_), individual anaerobic threshold (LT_2_), maximal lactate steady state (MLSS), or critical power (CP). Exercising in intensity domains relative to these thresholds (“moderate”: less than LT_1_; “heavy”: between LT_1_ and MLSS/CP; “severe”: above MLSS/CP) result in different levels of metabolic stimulus and time-dependent relationships for exercise tolerance and fatigue [6]. Large heterogeneity in the %V̇O_2peak_ and %HR_peak_ response at metabolic thresholds demonstrates that using these methods could result in markedly different metabolic stimuli between the individuals and serves as a significant limitation in the method [7]. However, the invasive and time-consuming nature of performing accurate exercise testing, reliability of the methods involved in calculating metabolic thresholds, and questions over whether certain threshold concepts accurately reflect intensity domain transitions [8], may challenge the use of these thresholds, as well as %V̇O_2peak_, for exercise prescription purposes. In addition, the cost of equipment and requirement for trained individuals to conduct tests creates further challenges for implementing these concepts. As such, there appears no consensus on how to best prescribe a relative exercise intensity that is homogenous across a large group of people.

An alternative that could be useful for large scale intervention, due to the ease of implementation, is to use ratings of perceived exertion (RPE). The RPE at LT_1_ has been shown to be independent of age, sex, training status [9], and mode of exercise [10]. This raises the potential of RPE being a simple method for prescribing a homogenous relative exercise intensity. However, this research is limited to studies of AB individuals performing treadmill running or cycle ergometry [9, 10]. It remains to be demonstrated how these findings may relate to upper body exercise modes, and to participants with SCI. Though RPE have been widely used to prescribe the intensity during training interventions [11], more evidence is required on the validity and reliability of RPE in the population with SCI [12].

One limitation to the use of RPE lies in the existence of different scales. The original, Borg’s RPE scale [13], is a 15-point scale ranging from 6 (no exertion) to 20 (maximal exertion) and results in linear relationships with markers of exercise intensity in AB [14] and SCI [15]. Whereas the Category Ratio 10 (CR10) scale ranges from 0 to 10 and results in nonlinear growth functions in SCI [16]. Borg’s RPE scale [17], and the CR10 [11] have been used in individuals with SCI to prescribe and regulate exercise intensity. However, it is difficult to assimilate results between studies using the different scales due to the lack of an evidence-based comparison. An original transformation table was produced to show the corresponding values on Borg’s RPE scale and the CR10 [18], though this was seemingly done based on the theoretical relationship of the common verbal anchors used by the scales. Only a single study has sought to apply statistical modelling in order to compare Borg’s RPE scale with the CR10 [19]. However, this was performed in AB adults performing lower body exercise, so this cannot be generalised to adults with SCI performing upper body exercise. Given the potential use of RPE for exercise intensity prescription in individuals with SCI, there is a need to investigate the relationship between RPE rated on different scales in this population in order to aid the interchangeable use of scales.

As such, there were two aims of this study. The first was to compare RPE on the CR10 with RPE on Borg’s RPE scale in AB participants during upper and lower body exercise, and in participants with SCI during upper body exercise only. The second aim was to investigate the RPE at the LT_1_ and LT_2_ within these exercise settings and populations. It was hypothesised that the two scales would show a strong relationship and that the RPE at LT_1_ and LT_2_ would be independent of exercise mode and participant group.

## Methods

### Experimental design

Twenty-four healthy adults volunteered to take part and provided written, informed consent. All procedures were approved by the Loughborough University ethics approvals human participants sub-committee; and the Local Ethics Committee of the Center for Human Movement Sciences, University Medical Center Groningen. Participants formed three, equally sized subgroups: AB who were recreationally active, but untrained in upper body endurance exercise; recreationally active people with paraplegia (PARA); and trained wheelchair rugby players with tetraplegia (TETRA), see Table 1. AB performed two exercise testing sessions in a randomised manner, one cycle ergometry (AB-CYC) and the other handcycle ergometry (AB-HC), separated by 2–7 days. Previously, reliable peak handcycle responses have been found without familiarisation in AB unaccustomed with upper body exercise [20]. PARA and TETRA each performed a single testing session using handcycling and wheelchair propulsion, respectively. These were performed as they were the main sporting activity performed by participants in the respective groups so increased the likelihood of valid and reliable responses. Cycle and handcycle tests were performed using a Cyclus 2 ergometer (Avantronic Richter, Leipzig, Germany) with the bike/handcycle attached, whilst wheelchair propulsion was performed on a motorised treadmill (HP Cosmos, Traunstein, Germany). All AB used the same bike (Viking Race 700c) and adjustable handcycle rig (Schmicking Reha-Technik GmbH, Holzwickede, Germany), whilst PARA and TETRA used their own handcycle and wheelchair rugby chair, respectively. Testing took place in two testing centres dependent on the location of the investigators (Peter Harrison Centre for Disability Sport, Loughborough University, UK; and Amsterdam Rehabilitation Research Center | Reade, Amsterdam, the Netherlands). AB and TETRA were tested in one location (UK), whilst PARA were tested in the other (the Netherlands). At each respective location, the same investigators performed all testing.

**Table 1:** Participant characteristics by group.

	Group		
	Able-bodied	Paraplegia	Tetraplegia
Sex (M/F)	8/0	7/1	7/1
Age (years)	21 (3)	47 (15)^a^	31 (7)
Height (m)	1.85 (0.07)	1.78 (0.05)	1.76 (0.11)
Body mass (kg)	79.4 (7.7)	74.5 (10.7)	68.3 (11.5)
Neurological level of injury	–	T4-L2	C5–C7
AIS	–	A = 4, C = 3, D = 1	A = 6, C = 2
Time since injury (years)	–	15 (19)	13 (7)

Data are presented as mean (standard deviation).

*AIS* American spinal injury association Impairment Scale.

^a^Significantly greater than other groups, *P* < 0.05.

### Graded exercise testing

Prior to testing, participants were presented with Borg’s RPE scale and the CR10 and read standard instructions on how to anchor their responses using the two scales [13]. They were instructed when rating their exertion, to focus on how hard, heavy and strenuous the physical task was, and not on any sensations of pain or discomfort [21].

Following a 5 min self-selected warm-up, participants completed an individualised continuous exercise test comprising 3 min stages. Starting workload and increment were 50 + 15 W for AB-CYC, 10 + 10 W for AB-HC, 20–45 + 20 W for PARA, and 1.2–1.7 + 0.2 m · s^−1^ for TETRA. V̇O_2_, ventilation (V̇E) and respiratory exchange ratio (RER), via online gas analysis (Metalyzer 3B, Cortex, Leipzig, Germany), as well as HR (RS400, Polar, Kempele, Finland) were monitored continuously. RPE were verbally reported in the final minute of each stage. One scale was presented with 45 s left in the stage, and the other with 15 s remaining. Order of scale presentation was consistent within but randomised between participants. Only the scale of interest was visible when reporting was required, all other data was blinded from participants throughout. A capillary blood sample from the earlobe was obtained in the final 30 s of each stage for determining blood lactate concentration ([BLa]). For AB and TETRA this was done using Biosen C-line (EKF Diagnostics, Barleben, Germany) and for PARA using Lactate Pro 2 (Arkray Factory Inc, FDK Corporation, Siga, Japan). For AB and TETRA, the test was terminated when [BLa] exceeded 4 mmol · L^−1^, or when 17 was reported on Borg’s RPE scale. This criteria was applied for TETRA, where they may have a blunted response in terms of [BLa]. PARA continued until volitional exhaustion.

AB and TETRA then received 15 min of either low intensity active recovery, or complete rest before performing a graded exercise test to exhaustion. Starting workload was set to the workload from the preceding test when [BLa] increased (0.5 mmol · L^−1^) above rest. Starting workload and increment were 110–180 W + 15 W · min^−1^, 30–60 W + 10 W · min^−1^ and 1.3–2.0 + 0.1 m · s^−1^ · min^−1^ for AB-CYC, AB-HC, and TETRA, respectively. Gas exchange variables and HR were collected throughout, with RPE and [BLa] immediately measured post-test.

Peak workload (PO or speed) was calculated based on the final completed stage and the proportion of any started, but not completed stage using the formula: Peak workload = F + [(t ÷ d) × I] where F = workload of final completed stage; t = time (s) spent in final, uncompleted stage; d = stage duration (s); and I = the workload increment. Gas exchange and HR data were subjected to a 30 s rolling average reported every 1 s, with the single greatest value taken as the peak response. The LT_1_ was identified as the intersection of the horizontal and ascending portions of the plot of log-[BLa] against log-V̇O_2_ [22]. The LT_2_ was identified as the [BLa] equal to 1.5 mmol · L^−1^ greater than LT_1_ [23]. The inverse of the log-V̇O_2_ at these points was recorded as the V̇O_2_ at LT_1_ and LT_2_. RPE on both scales were individually fit against [BLa] using a quadratic function (*ax*^2^ + b*x* + c; where *x* = [BLa]) for each participant. The resultant coefficients were subsequently used to calculate the RPE at LT_1_ and LT_2_.

### Statistical analyses

Analyses were performed using IBM SPSS Statistics Version 23.0 (IBM Corp., Armonk, NY) and MLWiN version 3.02 [24]. Data are presented as mean (standard deviation) with statistical significance accepted at *P* < 0.05. Data were checked for normal distribution using the Shapiro Wilk statistic. Where appropriate, standardised effect sizes (ES) were calculated to describe the magnitude of differences and categorised as trivial (<0.2), small (0.2–0.6), moderate (0.6–1.2), large (1.2–2.0), and very large (>2.0) [25].

First, explorative analyses per individual participant were performed, where curve analysis was used to compare the RPE values between scales. In each case the RPE on Borg’s RPE scale served as the independent variable, with RPE on CR10 the dependent variable. RPE on the CR10 was fit using linear (*y* = *ax* + b), quadratic (*y* = *ax*^2^ + b*x* + c), exponential (*y* = a × *e*^*bx*^) and power (*y* = a × *x*^b^) functions, where in each case *x* = RPE on Borg’s RPE scale and *y* = RPE on CR10. *F* tests were used to identify if the models reached statistical significance. In cases where all models were significant, the model with the greatest coefficient of determination (*R*^2^) was used for subsequent analysis. Second, a two-level random-intercept multilevel model was generated for each group, based on the model with the greatest *R*^2^ from the initial analysis. The models were multilevel to be able to adjust for the dependency of observations (i.e. number of stages in the graded exercise test) within participants. The regression models were created with stages as the first level and participant as the second level.

Differences in peak exercise responses between groups were assessed via one-way analysis of variance (ANOVA) with post-hoc Bonferroni correction for multiple comparisons. Similarly, differences between groups in V̇O_2_ (L · min^−1^, ml · kg^−1^ · min^−1^, %V̇O_2peak_) and RPE at LT_1_ and LT_2_ were also assessed via one-way ANOVA with post-hoc Bonferroni correction.

## Results

Peak exercise responses are shown in Table 2. Absolute V̇O_2peak_ was significantly greater in AB-CYC compared to AB-HC (*P* < 0.005; ES = 2.0), PARA (*P* < 0.005; ES = 1.9) and TETRA (*P* < 0.005; ES = 3.1). There was no significant difference in absolute V̇O_2peak_ between AB-HC and PARA (*P* > 0.995; ES = 0.2), AB-HC and TETRA (*P* = 0.12; ES = 1.4), or PARA and TETRA (*P* = 0.06; ES = 1.7), though in the latter two cases the ES were “large”. Similarly, relative V̇O_2peak_ was significantly greater in AB-CYC compared to AB-HC (*P* < 0.005; ES = 2.1), PARA (*P* < 0.005; ES = 1.9) and TETRA (*P* < 0.005; ES = 3.1). There was no significant difference in relative V̇O_2peak_ between AB-HC and PARA (*P* > 0.995; ES = 0.5), AB-HC and TETRA (*P* = 0.38; ES = 1.0), or PARA and TETRA (*P* = 0.06; ES = 1.6), though in the latter two cases the ES were “moderate” and “large” respectively.

**Table 2:** Peak exercise responses by group.

	AB-CYC	AB-HC	PARA	TETRA
V̇O_2_ (L · min^−1^)	3.81 (0.89)^a,b,c^	2.32 (0.58)	2.44 (0.53)	1.51 (0.59)
V̇O_2_ (ml · kg^−1^ · min^−1^)	47.99 (9.32)^a,b,c^	29.43 (7.90)	32.76 (6.05)	21.95 (7.36)
HR (beats · min^−1^)	185 (10)^c^	169 (12)^c^	179 (12)^c^	125 (11)
RER	1.17 (0.21)	1.27 (0.22)	1.24 (0.07)	1.23 (0.15)
V̇E (L · min^−1^)	137.1 (12.5)^a,c^	83.8 (19.0)	118.7 (32.0)^a,c^	53.3 (15.5)
[BLa] (mmol · L^−1^)	9.02 (0.82)^b,c^	8.56 (1.28)^b,c^	13.53 (3.06)^c^	4.56 (0.74)
RPE (Borg’s RPE)	19 (1)	19 (2)	20 (0)	19 (1)
RPE (CR10)	9 (1)	9 (2)	10 (0)	10 (1)
Power output (W)	259 (33)	128 (15)	150 (30)	–
Speed (m · s^−1^)	–	–	–	2.5 (0.4)

Data are presented as mean (standard deviation).

*[BLa]* blood lactate concentration, *CR10* Category Ratio 10, *HR* heart rate, *RER* respiratory exchange ratio, *RPE* rating of perceived exertion, *V̇E* minute ventilation, *V̇O2* oxygen uptake.

^a^Significantly different vs AB-HC.

^b^Significantly different vs PARA.

^c^Significantly different vs TETRA, *P* < 0.05.

### Comparison of RPE on CR10 with RPE on Borg’s RPE scale

Figures displaying the individual participant raw data comparing RPE on CR10 with Borg’s RPE Scale for all groups can be found in the Supplementary Material. Though in each group, all modelled functions (linear, quadratic, exponential, and power) were significant, coefficient of determination was always greatest when using a quadratic function (Table 3). Thus, the subsequent follow-up multilevel analysis also utilised a quadratic function. The quadratic multilevel modelling resulted in the following equations where in each case *x* = RPE on Borg’s RPE Scale and *y* = RPE on CR10:

$$AB\!-\!CYC\!:y = 0.023x^2 + 0.067x - 0.754\;(R^2 = 0.970)$$

$$AB\!-\!HC\!:y = 0.024x^2 + 0.085x - 1.087\;(R^2 = 0.968)$$

$$PARA\!:y = 0.019x^2 + 0.240x - 2.212\;(R^2 = 0.965)$$

$$TETRA\!:y = 0.015x^2 + 0.306x - 1.989\;(R^2 = 0.967)$$

**Table 3:** Group-averaged coefficient of determination for each model of RPE on CR10 against RPE on Borg’s RPE scale.

Group	*R*^2^			
	Linear	Quadratic	Exponential	Power
AB-CYC	0.949 (0.025)	0.974 (0.019)	0.900 (0.033)	0.933 (0.032)
AB-HC	0.951 (0.031)	0.979 (0.018)	0.881 (0.058)	0.920 (0.064)
PARA	0.971 (0.013)	0.979 (0.012)	0.923 (0.029)	0.966 (0.018)
TETRA	0.966 (0.032)	0.984 (0.011)	0.920 (0.041)	0.957 (0.029)

Data are presented as mean (standard deviation).

Using the above formulae, transformed values were calculated and are displayed in Table 4.

**Table 4:** Borg’s RPE scale and proposed transformed values of RPE on the CR10.

Borg’s RPE value	Transformed value on CR10			
	AB-CYC	AB-HC	PARA	TETRA
6	0.5	0.5	0	0.5
7	1	0.5	0.5	1
8	1	1	1	1
9	2	2	2	2
10	2	2	2	3
11	3	3	3	3
12	3	3	3	4
13	4	4	4	5
14	5	5	5	5
15	5	6	6	6
16	6	6	7	7
17	7	7	7	8
18	8	8	8	8
19	9	9	9	9
20	10	10	10	10

*CR10* Category Ratio 10, *RPE* rating of perceived exertion.

### Responses at LT_1_ and LT_2_

**Fig. 1:**
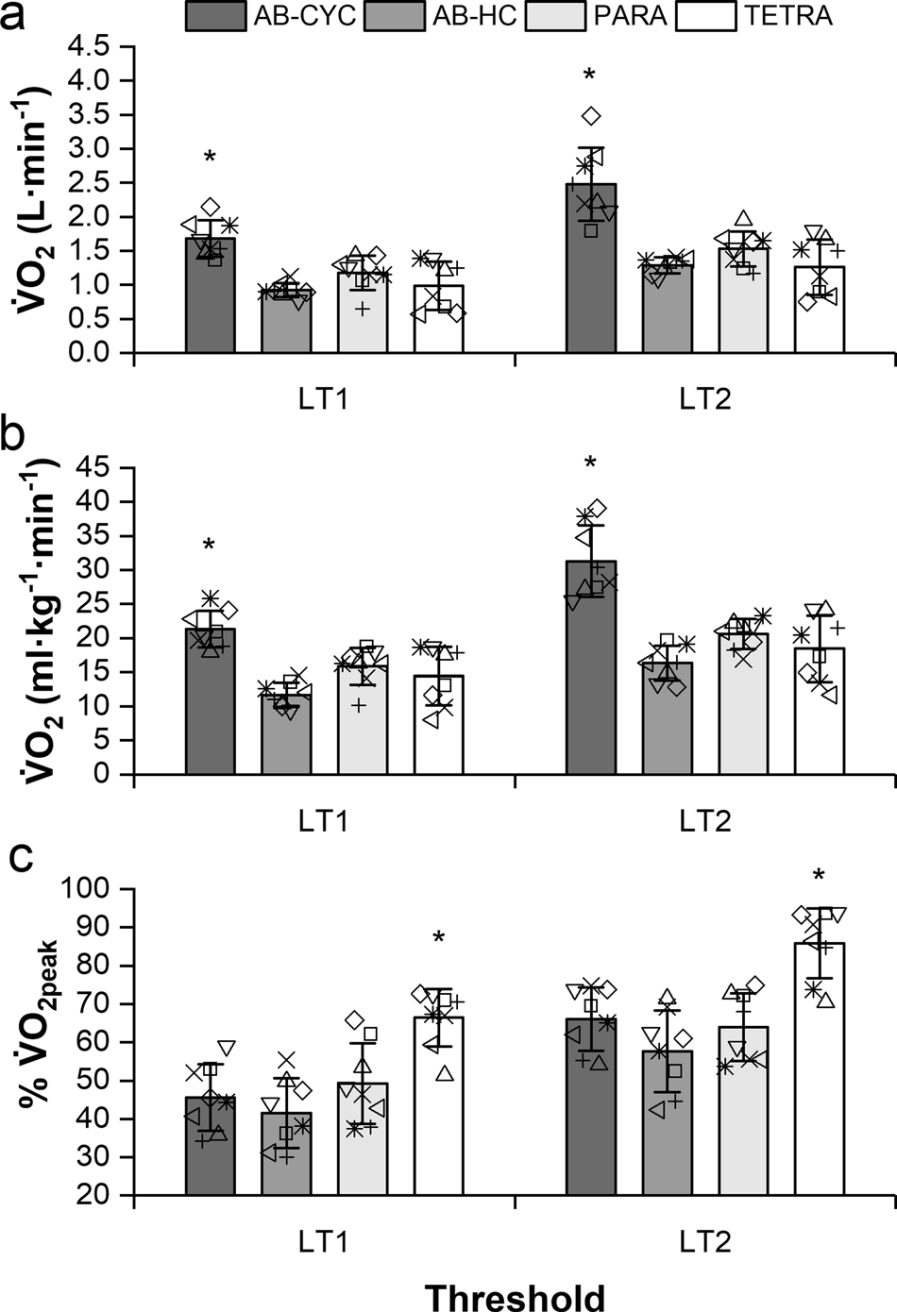
Group responses for **a** absolute V̇O_2_, **b** relative V̇O_2_, and **c** percentage of V̇O_2peak_ at lactate thresholds. Data are presented as mean (standard deviation) with individual points overlaid. Asterisk indicates significantly greater than other groups, *P* < 0.0005.

At LT_1_, absolute V̇O_2_ was significantly greater in AB-CYC compared to AB-HC (0.76, 0.39–1.13 L · min^−1^; *P* < 0.005; ES = 3.8), PARA (0.51, 0.13–0.88 L · min^−1^; *P* < 0.005; ES = 1.9) and TETRA (0.70, 0.32–1.07 L · min^−1^; *P* < 0.005; ES = 2.2; Fig. 1a). Similarly, relative V̇O_2_ at LT_1_ was significantly greater in AB-CYC compared to AB-HC (9.59, 5.31–13.86 ml · kg^−1^ · min^−1^; *P* < 0.005; ES = 4.2), PARA (5.44, 1.17–9.71 ml · kg^−1^· min^−1^; *P* = 0.01; ES = 2.0) and TETRA (6.87, 2.59–11.14 ml · kg^−1^ · min^−1^; *P* < 0.005; ES = 1.9; Fig. 1b). Conversely, the %V̇O_2peak_ at LT_1_ was significantly greater in TETRA compared to AB-CYC (20.9, 8.1–33.7%; *P* < 0.005; ES = 2.6), AB-HC (25.0, 12.1–37.8%; *P* < 0.005; ES = 3.0) and PARA (17.2, 4.4–30.0%; *P* < 0.005; ES = 1.9; Fig. 1c).

Absolute V̇O_2_ at LT_2_ was significantly greater in AB-CYC compared to AB-HC (1.20, 0.68–1.71 L · min^−1^; *P* < 0.005; ES = 3.1), PARA (0.95, 0.43–1.47 L · min^−1^; *P* < 0.005; ES = 2.3) and TETRA (1.22, 0.70–1.74 L · min^−1^; *P* < 0.005; ES = 2.6; Fig. 1a). Relative V̇O_2_ at LT_2_ was also significantly greater in AB-CYC than AB-HC (14.94, 9.35–20.52 ml · kg^−1^ · min^−1^; *P* < 0.005; ES = 3.6), PARA (10.70, 5.11–16.28 ml · kg^−1^ · min^−1^; *P* < 0.005; ES = 2.7) and TETRA (12.86, 7.27–18.44 ml · kg^−1^ · min^−1^; *P* < 0.005; ES = 2.6; Fig. 1b). At LT_2_ the %V̇O_2peak_ was significantly greater in TETRA than AB-CYC (19.9, 6.7–33.1%; *P* < 0.005; ES = 2.3), AB-HC (28.3, 15.1–41.4%; *P* < 0.005; ES = 2.8) and PARA (22.0, 8.8–35.1%; *P* < 0.005; ES = 2.4; Fig. 1c).

**Fig. 2:**
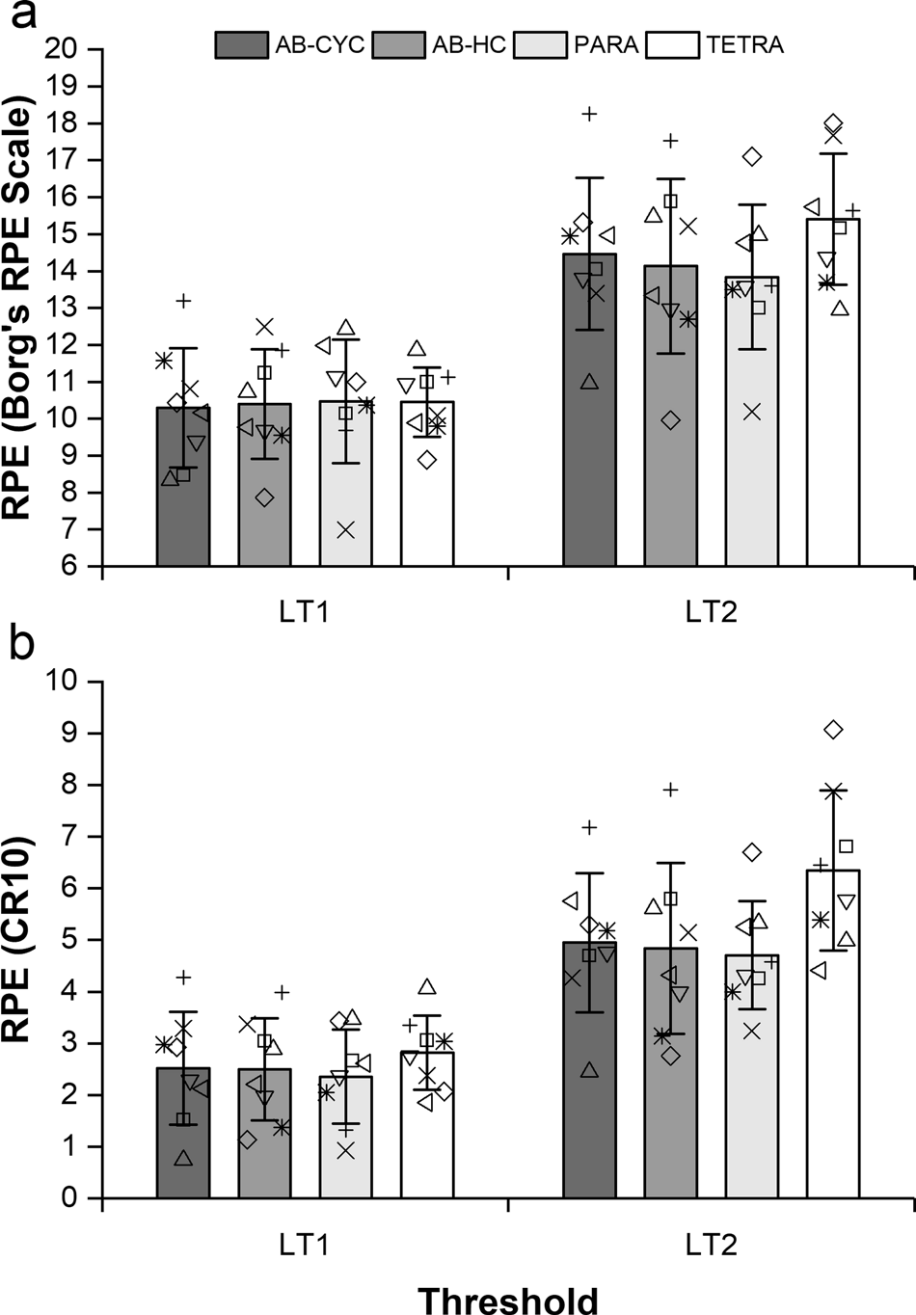
Group responses for RPE at lactate thresholds on **a** Borg’s RPE scale and **b** CR10. Data are presented as mean (standard deviation) with individual points overlaid.

There was no significant effect of group for the RPE on Borg’s RPE scale (*F*_(3)_ = 0.02, *P* = 0.99; *F*_(3)_ = 0.86, *P* = 0.47) (Fig. 2a) or CR10 (*F*_(3)_ = 0.36, *P* = 0.78; *F*_(3)_ = 2.34, *P* = 0.10) (Fig. 2b) at LT_1_ or LT_2_, respectively.

## Discussion

This is the first study to directly compare Borg’s RPE scale and CR10 in participants with SCI during upper body exercise, as well as AB during upper and lower body exercise. This was with a view to helping inform the use of RPE for exercise intensity prescription purposes. The principle finding was of strong association between the two scales independent of exercise mode or population group, as shown by the high coefficients of determination (Table 3). The strong association indicates that Borg’s RPE scale and CR10 can be used interchangeably, with the resultant transformation table acting as a reference for prescribing, or interpreting, equivalent ratings.

When modelling exertion from Borg’s RPE scale with the CR10, a quadratic function was found to explain the greatest amount of variation between scales. The quadratic coefficients found for each measure of RPE (range 0.015–0.024) are similar to those found previously when comparing Borg’s RPE scale with CR10 in young, healthy AB adults during incremental (0.020) and interval-based (0.034) cycle ergometry [19]. Indeed, applying a quadratic function to the original, proposed transformation by Borg and Ottoson [18] results in a quadratic coefficient of 0.041. Importantly, this study not only shows that the relationship between Borg’s RPE scale and CR10 is similar in AB adults performing lower and upper body exercise, but also in participants with paraplegia and tetraplegia. Despite the potential use of RPE for regulating exercise intensity in participants with SCI [17], there is still little evidence supporting the validity and reliability of doing so [12]. Specifically, in the review of van der Scheer et al. it was noted that though the CR10 is often used to prescribe intensity during training interventions in SCI, evidence supporting validity and reliability of RPE in SCI all came from studies using Borg’s RPE scale. It is possible that the CR10 has proven popular as it can also be used to calculate a training load through the use of the session RPE [26]. Nevertheless, this study can, for the first time, provide researchers and practitioners with specific transformation tables to be able to equate RPE between Borg’s RPE scale and CR10, and to be used as part of exercise intensity prescription in participants with paraplegia and tetraplegia.

A further finding from the current study was that RPE at the LT_1_ and LT_2_ was independent of exercise modality and the presence/level of SCI. This is despite differences in the absolute V̇O_2_ and percentage of V̇O_2peak_ between groups at which the LT_1_ and LT_2_ occurred. This provides support for RPE as a potential method for prescribing a training intensity with a homogenous metabolic stimulus across population groups. Average RPE at LT_1_ was equal for AB-CYC, AB-HC, and TETRA, being 10 (2) on Borg’s RPE scale and 3 (1) on the CR10. For PARA, RPE at LT_1_ was 11 (2) on Borg’s RPE scale and 2 (1) on the CR10. Median (interquartile range) RPE at LT_1_ in athletes with paraplegia and tetraplegia have been found to be 12 (11–13) and 13 (12–14), respectively, on Borg’s RPE scale [27]. Whilst in AB participants performing lower body exercise, LT_1_ has previously been found to occur at RPE of 10 (2) [9], 11 (2) [10], and between 13–14 [28] on Borg’s RPE scale, and at 3 (1) on the CR10 [29]. Though, generally, the results of the present study appear to confirm previous findings there are important considerations that need to be made. Differences exist in the methods used to identify the LT_1_ between studies. Whilst a log-log approach, as in this study, has been used [27, 29], the LT_1_ has also been identified as corresponding to the intensity just prior to a curvilinear increase in [BLa], often through visual inspection [28]. It is possible that variability associated with visual inspection methods could lead to the observed differences in RPE at LT_1_ between studies.

Notwithstanding the variation caused by identification methods, there are also inter-individual differences in the RPE at both LT_1_ and LT_2_. This variation is particularly significant when considering whether RPE is suitable to be used for exercise intensity prescription. In the present study the standard deviation for RPE at LT_1_ ranged between 1 and 2 units for both Borg’s RPE scale and the CR10, which is similar to that found in a previous study [27]. The level of inter-individual variation was also similar between groups, suggesting that this was not affected by level of SCI, or fitness. Though seemingly small, it still remains that prescribing the same RPE (i.e. 11 on Borg’s RPE scale) to two individuals could conceivably result in one exercising above, and the other below, their LT_1_. In principle, this precludes from the ability to unilaterally prescribe a specific RPE for a population-wide homogenous exercise intensity prescription. Much akin to the criticism that has been made of fixed %V̇O_2peak_ [7]. However, this needs to be applied within the specific context of the person involved. For the general population with SCI trying to meet the exercise guidelines [5], precise control of the exercise intensity may not be as important, so RPE of 11 (2 on CR10) for paraplegia and 10 (3 on CR10) for tetraplegia can be recommended for exercising at LT_1_. This could potentially aid the implementation of home-based exercise programmes. In contrast, athletes for whom specific adaptations are desired, it would still be preferable to understand the individual relationship between RPE, V̇O_2_ and [BLa] in order to tailor their training prescription accordingly.

Despite the promising results, this study does have some methodological limitations. Firstly, groups were not matched for age or fitness level, whilst there were no sedentary or untrained participants with SCI. However, results supporting the aims of the study were not significantly different between groups, suggesting that not matching has not had an impact on the findings. The protocol for eliciting peak exercise responses was also not consistent between the testing sites. This would have had no impact on the submaximal results, as both sites used 3 min stages for this purpose. It is possible that peak responses could have been affected by the difference in protocol design. However, it has been shown that V̇O_2peak_ is similar in protocols with significantly different durations [30], so we do not feel that this has impacted our findings.

In conclusion, this study showed that there was a high level of association between RPE when rated on Borg’s RPE scale and CR10 in AB participants and those with paraplegia and tetraplegia. The RPE at LT_1_ and LT_2_ was independent of mode of exercise and level of SCI. It is possible that using RPE could serve as a simple method for prescribing exercise intensity, with the transformation table able to aid interchangeable use of Borg’s RPE scale and CR10. However, inter-individual variation precludes from making firm recommendations about the use of RPE for prescribing a homogenous exercise intensity between individuals.

## Supplementary information

Supplementary material

## Data Availability

The datasets generated and analysed during the current study are available from the corresponding author on reasonable request.

## References

[1] Pedersen BK, Saltin B (2015). Exercise as medicine - evidence for prescribing exercise as therapy in 26 different chronic diseases. Scand J Med Sci Sports.

[2] van der Scheer JW, Martin Ginis KA, Ditor DS, Goosey-Tolfrey VL, Hicks AL, West CR (2017). Effects of exercise on fitness and health of adults with spinal cord injury. Neurology.

[3] Jamnick NA, Pettitt RW, Granata C, Pyne DB, Bishop DJ (2020). An examination and critique of current methods to determine exercise intensity. Sports Med.

[4] Riebe D, Ehrman JK, Liguori G, Magal M (2018). ACSM’s guidelines for exercise testing and prescription. Tenth edit.

[5] Martin Ginis KA, van der Scheer JW, Latimer-Cheung AE, Barrow A, Bourne C, Carruthers P (2018). Evidence-based scientific exercise guidelines for adults with spinal cord injury: an update and a new guideline. Spinal Cord.

[6] Burnley M, Jones AM (2018). Power–duration relationship: physiology, fatigue, and the limits of human performance. Eur J Sport Sci.

[7] Iannetta D, Inglis EC, Mattu AT, Fontana FY, Pogliaghi S, Keir DA (2020). A critical evaluation of current methods for exercise prescription in women and men. Med Sci Sports Exerc.

[8] Poole DC, Rossiter HB, Brooks GA, Gladden LB (2021). The anaerobic threshold: 50+ years of controversy. J Physiol.

[9] Rynders CA, Angadi SS, Weltman NY, Gaesser GA, Weltman A (2011). Oxygen uptake and ratings of perceived exertion at the lactate threshold and maximal fat oxidation rate in untrained adults. Eur J Appl Physiol.

[10] Scherr J, Wolfarth B, Christle JW, Pressler A, Wagenpfeil S, Halle M (2013). Associations between Borg’s rating of perceived exertion and physiological measures of exercise intensity. Eur J Appl Physiol.

[11] Pelletier CA, Totosy de Zepetnek JO, MacDonald MJ, Hicks AL (2015). A 16-week randomized controlled trial evaluating the physical activity guidelines for adults with spinal cord injury. Spinal Cord.

[12] van der Scheer JW, Hutchinson M, Paulson T, Martin Ginis KA, Goosey-Tolfrey VL (2018). Reliability and validity of subjective measures of aerobic intensity in adults with spinal cord injury: a systematic review. PMR.

[13] Borg GA (1998). Borg’s Perceived Exertion and Pain Scales.

[14] Chen MJ, Fan XT, Moe ST (2002). Criterion-related validity of the Borg ratings of perceived exertion scale in healthy individuals: a meta-analysis. J Sports Sci.

[15] Goosey-Tolfrey VL, Paulson T, Tolfrey K, Eston R (2014). Prediction of peak oxygen uptake from differentiated ratings of perceived exertion during wheelchair propulsion in trained wheelchair sportspersons. Eur J Appl Physiol.

[16] Au JS, Totosy de Zepetnek JO, MacDonald MJ (2017). Modeling perceived exertion during graded arm cycling exercise in spinal cord injury. Med Sci Sports Exerc.

[17] Paulson TA, Bishop NC, Leicht CA, Goosey-Tolfrey VL (2013). Perceived exertion as a tool to self-regulate exercise in individuals with tetraplegia. Eur J Appl Physiol.

[18] Borg G, Ottoson D (1986). The Perception of Exertion in Physical Work..

[19] Arney BE, Glover R, Fusco A, Cortis C, de Koning JJ, van Erp T (2019). Comparison of rating of perceived exertion scales during incremental and interval exercise. Kinesiology.

[20] Hutchinson MJ, Paulson TAW, Eston R, Tolfrey VLG (2017). Assessment of peak oxygen uptake during handcycling: Test-retest reliability and comparison of a ramp-incremented and perceptually-regulated exercise test. PLoS One.

[21] Marcora SM. Effort: Pereption of. In: Goldstein EB, editor. Encyclopedia of perception. Thousand Oaks, California: Sage; 2010. pp 380–3.

[22] Beaver WL, Wasserman K, Whipp BJ (1985). Improved detection of lactate threshold during exercise using a log-log transformation. J Appl Physiol.

[23] Dickhuth H-H, Huonker M, Münzel T, Drexler H, Berg A, Keul J. Individual anaerobic threshold for evaluation of competitive athletes and patients with left ventricular dysfunction. In: Bachl N, Graham TE, Lollgen H, editors. Advances in Ergometry. Berlin Heidelberg: Springer; 1991. pp 173–9.

[24] Charlton C, Rasbash J, Browne WJ, Healy M, Cameron B. MLwiN Version 3.05. Centre for Multilevel Modelling, University of Bristol. 2020.

[25] Batterham AM, Hopkins WG (2006). Making meaningful inferences about magnitudes. Int J Sports Physiol Perform.

[26] de Groot S, Hoekstra SP, Grandjean Perrenod Comtesse P, Kouwijzer I, Valent LJ (2018). Relationship between internal and external handcycle training load in people with spinal cord injury training for the handbikebattle. J Rehabil Med.

[27] Leicht CA, Griggs KE, Lavin J, Tolfrey K, Goosey-Tolfrey VL (2014). Blood lactate and ventilatory thresholds in wheelchair athletes with tetraplegia and paraplegia. Eur J Appl Physiol.

[28] Demello J, Cureton K, Boineau R, Singh M (1987). Ratings of perceived exertion at the lactate threshold in trained and untrained men and women. Med Sci Sports Exerc.

[29] Abe D, Yoshida T, Ueoka H, Sugiyama K, Fukuoka Y. Relationship between perceived exertion and blood lactate concentrations during incremental running test in young females. BMC Sports Sci Med Rehabil. 2015;7. 10.1186/2052-1847-7-5. 10.1186/2052-1847-7-5 PMC4429818 25973209

[30] Midgley AW, Bentley DJ, Luttikholt H, McNaughton LR, Millet GP (2008). Challenging a dogma of exercise physiology: does an incremental exercise test for valid VO2max determination really need to last between 8 and 12 min?. Sports Med.

