# Supplementary material for: Comparison of two Borg exertion scales for monitoring exercise intensity in able-bodied participants, and those with paraplegia and tetraplegia
Source: Spinal Cord. 2021 May 26;59(11):1162–9. doi: 10.1038/s41393-021-00642-4 (PMC8560635; doi:10.1038/s41393-021-00642-4)
Supplement: Supplementary file 1 — Supplementary material [file 41393_2021_642_MOESM1_ESM.pdf]

Hutchinson MJ, Kouwijzer I, de Groot S, Goosey-Tolfrey VL, Comparison of two Borg exertion scales for monitoring exercise intensity in able-bodied participants, and those with paraplegia and tetraplegia, Spinal Cord, 2021.

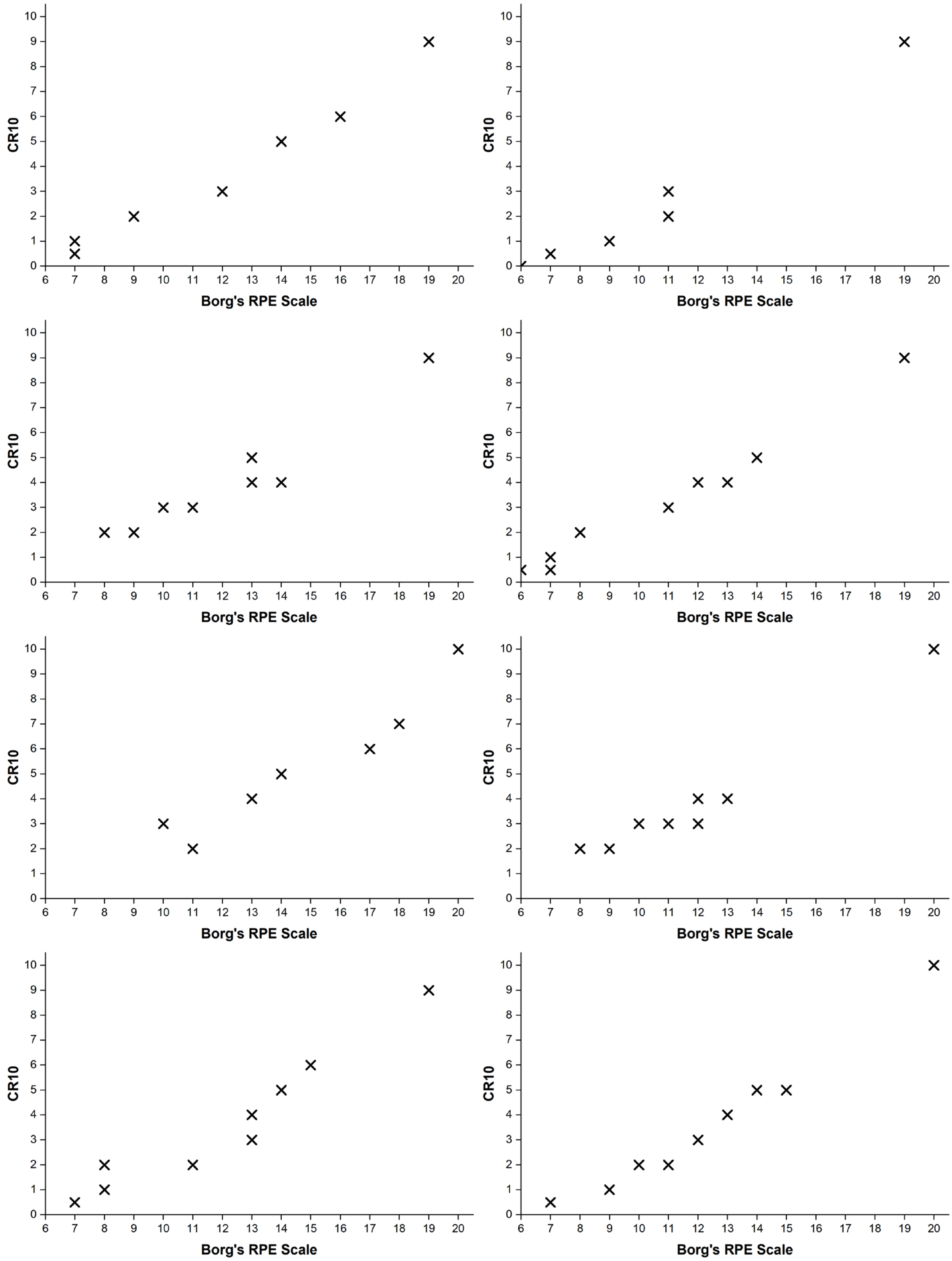

Supplementary Material 1: Raw rata comparing RPE on Borg's RPE Scale (6-20) and CR10 (0-10) for individual participants in AB-CYC. Each separate figure represents an individual participant.

Hutchinson MJ, Kouwijzer I, de Groot S, Goosey-Tolfrey VL, Comparison of two Borg exertion scales for monitoring exercise intensity in able-bodied participants, and those with paraplegia and tetraplegia, Spinal Cord, 2021.

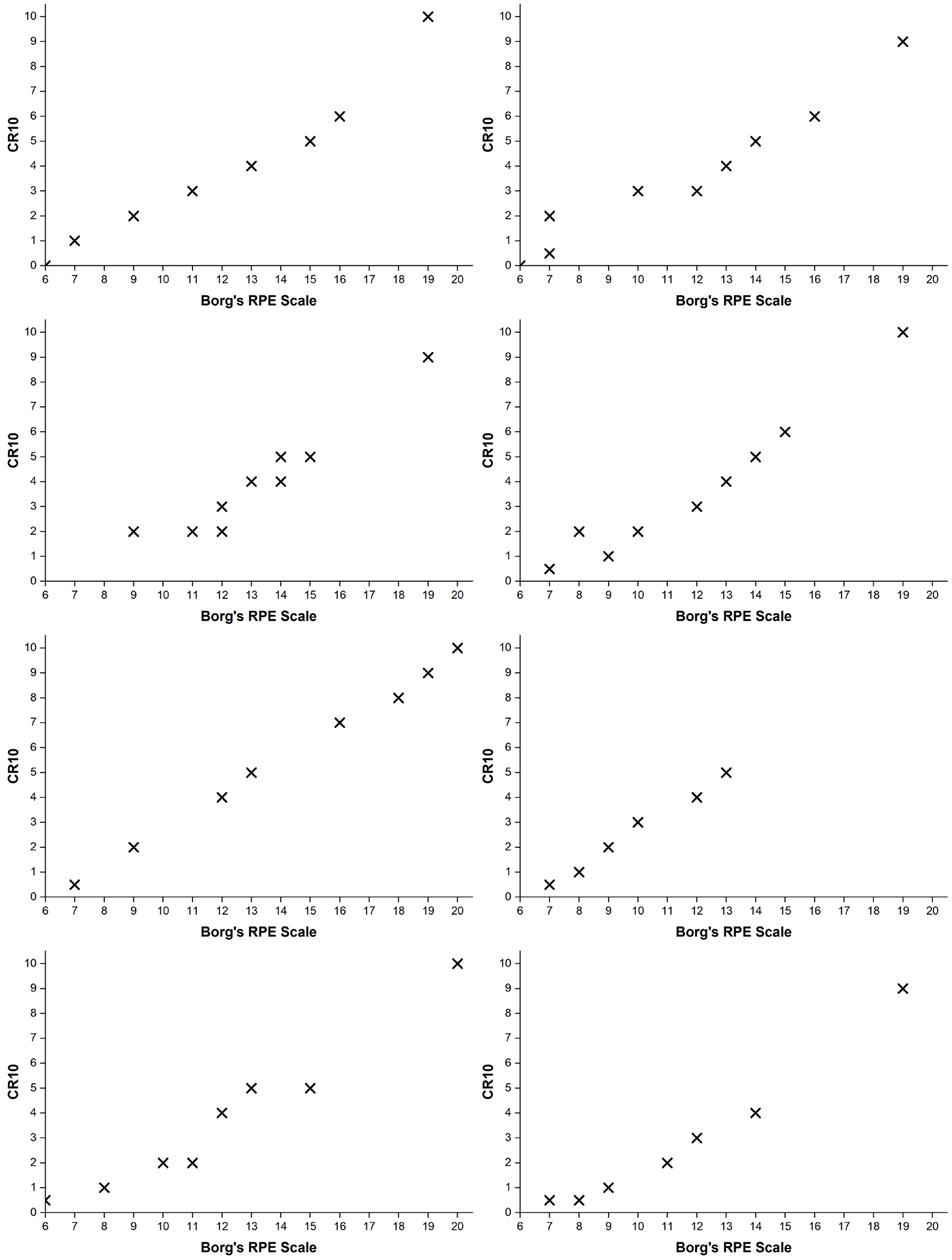

Supplementary Material 2: Raw rata comparing RPE on Borg's RPE Scale (6-20) and CR10 (0-10) for individual participants in AB-HC. Each separate figure represents an individual participant.

Hutchinson MJ, Kouwijzer I, de Groot S, Goosey-Tolfrey VL, Comparison of two Borg exertion scales for monitoring exercise intensity in able-bodied participants, and those with paraplegia and tetraplegia, Spinal Cord, 2021.

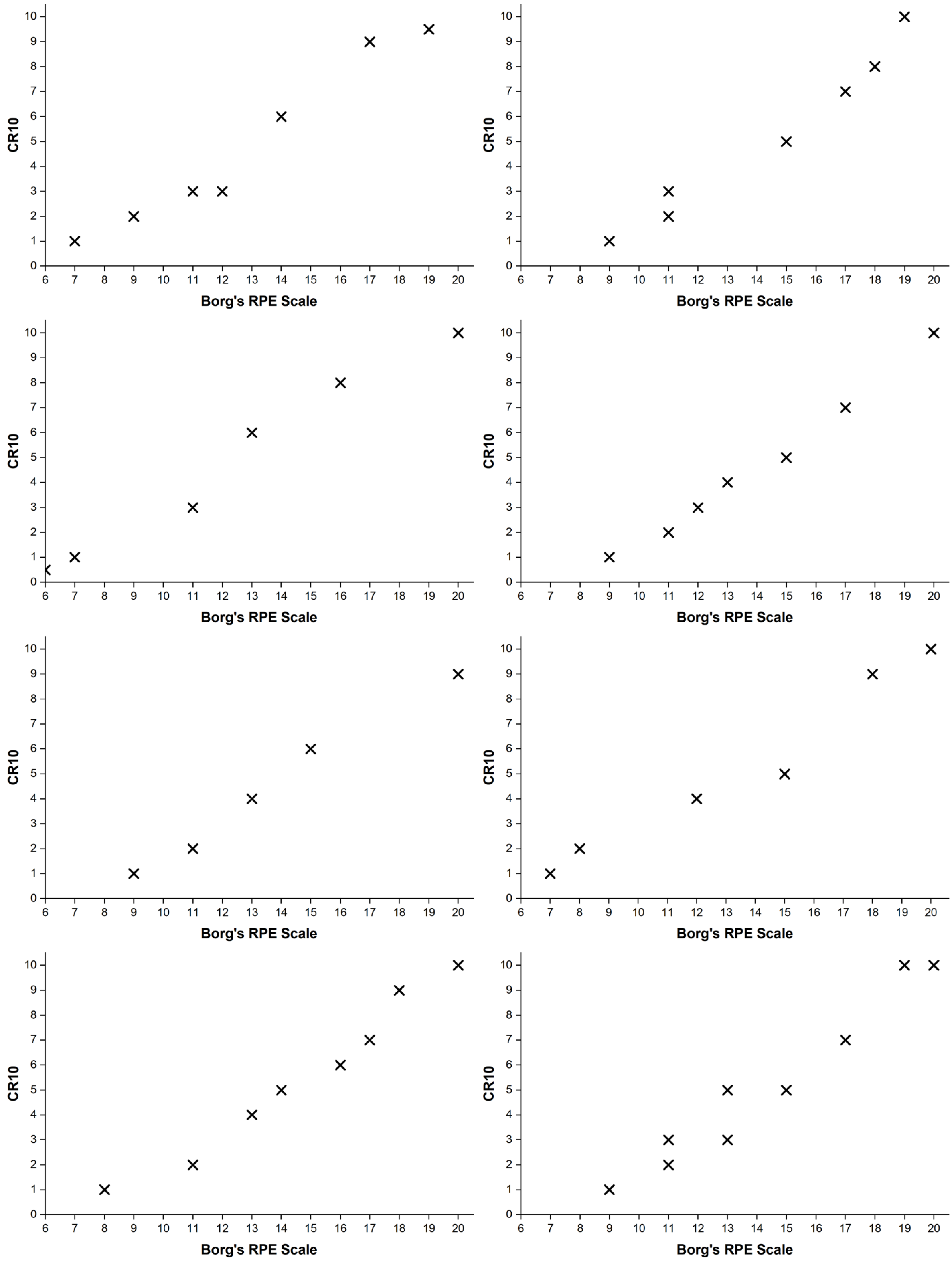

Supplementary Material 3: Raw rata comparing RPE on Borg’s RPE Scale (6-20) and CR10 (0-10) for individual participants in PARA. Each separate figure represents an individual participant.

Hutchinson MJ, Kouwijzer I, de Groot S, Goosey-Tolfrey VL, Comparison of two Borg exertion scales for monitoring exercise intensity in able-bodied participants, and those with paraplegia and tetraplegia, Spinal Cord, 2021.

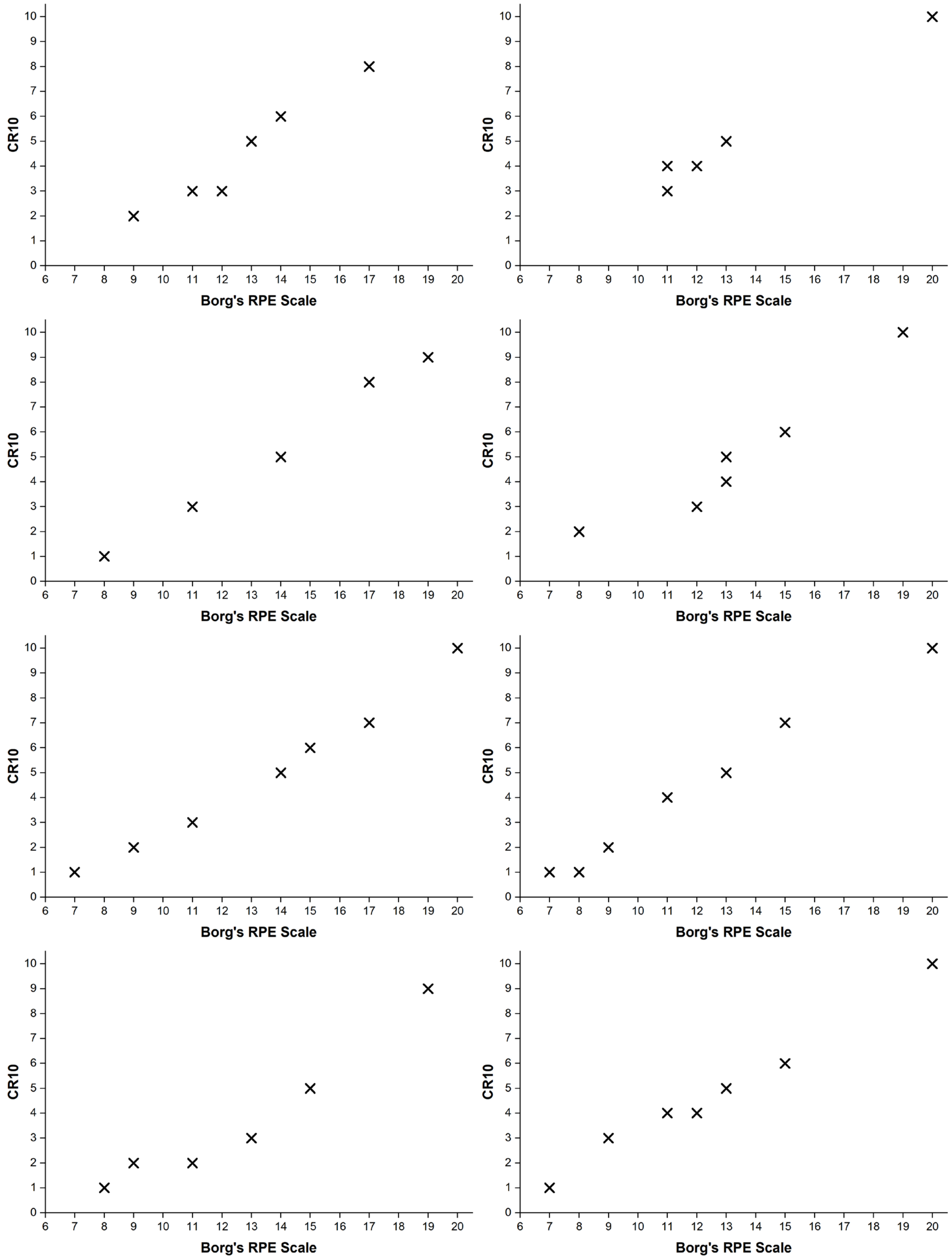

Supplementary Material 4: Raw rata comparing RPE on Borg’s RPE Scale (6-20) and CR10 (0-10) for individual participants in TETRA. Each separate figure represents an individual participant.
